# Correlation between fibronectin binding protein A expression level at the surface of recombinant *lactococcus lactis* and plasmid transfer *in vitro* and *in vivo*

**DOI:** 10.1186/s12866-014-0248-9

**Published:** 2014-09-25

**Authors:** Juliana F Almeida, Denis Mariat, Vasco Azevedo, Anderson Miyoshi, Alejandra de Moreno de LeBlanc, Silvina del Carmen, Rebeca Martin, Philippe Langella, Jean-Guy LeBlanc, Jean-Marc Chatel

**Affiliations:** INRA, UMR1319 Micalis, Domaine de Vilvert, F-78350 Jouy-en-Josas, France; AgroParisTech, UMR1319 Micalis, F-78350 Jouy-en-Josas, France; Institute of Biological Sciences, Federal University of Minas Gerais (UFMG-ICB), Belo Horizonte, MG Brazil; CERELA-CONICET, Chacabuco 145, San Miguel de Tucumán, Argentina

**Keywords:** FnBPA, Lactococcus lactis, nisA promoter, Caco-2 cells, BMDCs, Invasiveness, Plasmid transfer

## Abstract

**Background:**

Fibronectin Binding Protein A (FnBPA) is an invasin from *Staphylococcus aureus* that allows this pathogen to internalize into eukaryote cells. It was previously demonstrated that recombinant *Lactococcus lactis* expressing FnBPA were invasive and able to transfer a plasmid to eukaryotic cells *in vitro* and *in vivo.* In this study, the invasivity of recombinant strains of *Lactococcus lactis* that express FnBPA under the control of its constitutive promoter or driven by the strong nisin inducible expression system (NICE) were studied.

**Results:**

It was demonstrated that the *nisA* promoter allows an increase of FnBPA expression on the surface of *Lactococcus lactis* surface, as shown by flow cytometry, which subsequently enhanced internalization and plasmid transfer properties *in vitro* in Caco2 cells and Bone Marrow Dendritic Cells. *In vivo*, the use of *nisA* promoter increase the plasmid transfer in cells of both the small and large intestine of mice.

**Conclusion:**

FnBPA expression at the surface of recombinant *L. lactis* is positively correlated to internalization and DNA transfer properties. The recombinant strains of *L. lactis* that expresses FnBPA under the control of the nisin inducible expression system could thus be considered as an improved tool in the field of DNA transfer.

## Background

DNA delivery by lactic acid bacteria (LAB) is a topic that has been the focus of an increasing amount of research groups, not only for its potential use for vaccination purposes, but also as a mean to deliver bioactive compounds directly in the gastrointestinal tract [1-6]. In this regard, a promising advance has been the development of a system based on a new vector for DNA delivery using lactococci, named Vaccination using lactic acid bacteria or pValac [7]. This plasmid harbours an eukaryotic region containing the CytoMegaloVirus promoter (pCMV) under which the gene of interest can be expressed, the polyadenylation signal of Bovine growth hormone (BGH polyA) which is required for gene expression by eukaryotic host cells, a prokaryotic region containing the RepA/RepC replication origins for both *Escherichia coli* and *Lactococcus* (*L.*) *lactis*, and a chloramphenicol resistance gene (Cm) for bacteria selection. In order to allow expression of the genes of interest by eukaryotic cells, such as those of the gastrointestinal tract, these DNA delivery vectors must be effectively incorporated into these cells. An interesting way to achieve this latter objective is to take advantage of the mechanisms used by invasive pathogens.

Pathogenic bacteria can be internalized by eukaryote cells by using invasins expressed on their cell surface [8] such as the Fibronectin Binding Protein A (FnBPA) stemming from *Staphylococcus* (*S.*) *aureus* [9], the Internalin A from *Listeria monocytogenesis* [10,11], or the invasin of *Yersinia pseudotuberculosis* [12,13]. Thus, these proteins possess the potential to be used as tools in genetic engineering for the design of invasive bacterial strains developed from non-pathogenic strains considered “GRAS” (Generally Regarded As Safe) for human health, such as the model LAB, *L. lactis*.

In the particular case of FnBPA, the internalization process involves three molecules including the bacterial FnBPA, the fibronectin synthesized by the host, and a clustering of integrins from the host cell surface. The binding between these three entities results in the bacterial uptake by the eukaryote host involving tyrosine phosphorylation and actin rearrangement [14].

The role of FnBPA in cell internalization using *L. lactis* has been previously described by Que et al. [15,16]. This process has been used in the design of recombinant bacterial strains expressing FnBPA with varying results, thus we hypothesized that an increase of the external expression of the invasin could improve the functionality of the DNA delivery system. One method of accomplishing this goal would be to use a strong heterologous promoter such as the *nisA* gene promoter which has been described as being one of the most efficient promoters used during the last years [17,18]. The *nisA* promoter is derived from the *Lactococcus lactis* nisin gene cluster where nisin is considered as an outside inducer that requires the control of the *nisK* and *nisR* gene regulators. This strategy could improve current constructs and improve DNA delivery in eukaryote cells.

The objectives of this work were to construct a *Lactococcus lactis* strain expressing the FnBPA gene under the control of the *nisA* promoter and evaluate if this new construct was able to increase FnBPA expression compared to the use of constitutive FnBPA promoter and consequently improve bacterial internalization and nucleic acid transfer in eukaryotic cells *in vitro* and in the gastrointestinal tract of mice.

## Results

### pNisFnBPA plasmid design

Plasmid pNisFnBPA was obtained by placing the *fnbpA* gene of *S. aureus*, without its constitutive promoter, but under control of the *nisA* promoter (PnisA). The backbone of this construction is provided by the pOri253 plasmid allowing resistance to erythromicyn. The final plasmid pNisFnBPA was used to transform the *L. lactis* NZ9000 wild type strain, resulting in LL-pNisFnBPA strain, since this strain, a derivative of *L. lactis* MG1363, contains the *nisRK* gene necessary for the expression of genes downstream of the nisA promoter in the presence of sub inhibitory concentrations of nisin.

### FnBPA expression characterized by FACS analysis

**Figure 1:**
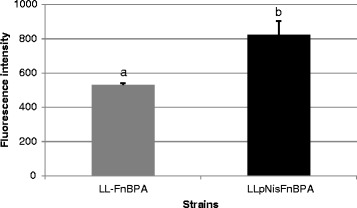
**FnBPA expression at the surface of LL-pNisFnBPA and LL-FnBPA as analyzed by flow cytometry where the secondary antibody was labeled with FITC.** Results are expressed as means ± standard deviation. ^a-b^Means with a different letter are significantly different from each other with a p ≤ 0.05.

FnBPA expression at the surface of LL-pNisFnBPA and LL-FnBPA was analyzed by fluorescence-activated cell sorting (FACS) using anti-FnBPA antibody after nisin induction. An increase of FnBPA expression in the range of 55% is observed in the strain with FnBPA expressed under the control of *nisA* promoter (Figure 1), compared to the strain with the constitutive promoter. Different concentrations of nisin were used for induction (0, 0.1, 1.0, 2.5, 5.0, and 10 ng/ml), and it was shown that the best results were obtained with the highest concentration used (10 ng/ml, data not shown) which is in accordance to the optimal concentrations for the expression of genes of interest in the description of this system [17].

### *In vitro* invasiveness assay in caco-2 cells and BMDCs

**Figure 2:**
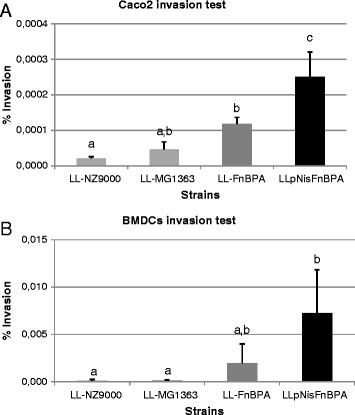
***In vitro*** **invasiveness assays of LL-NZ9000, LL-MG1363, LL-FnBPA and LL-PnisA-FnBPA strains in A) Caco-2 cells and B) BMDCs.** Results are expressed as means ± standard deviation. ^a-c^Means with a different letter are significantly different from each other with a p ≤ 0.05.

The recombinant strains LL-FnBPA and LL-pNisFnBPA were co-incubated with Caco2 cells or bone marrow derived dendritic cells (BMDCs), as well as two control *L. lactis* wild type strains (LL-NZ9000 and LL-MG1363). The bacterial invasiveness was then defined by the ratio between internalized bacteria, determined by colony forming units observed after cell lysis and plating, and bacteria input for co-incubation. Figure 2 shows the invasiveness levels obtained with the four *L. lactis* strains using Caco-2 and BMDC cellular models, respectively. In the Caco-2 cell model, it can be noted, that the invasive LL-FnBPA strain allows greater invasiveness levels (between 3 and 6 times) than the control strains. This invasiveness value can even reach a 10 fold increase in the case of BMDCs model. Moreover, in both cellular models, the LL-pNisFnBPA strain exhibits invasiveness levels 3 to 4 times greater than the LL-FnBPA strain. Therefore, the increase of FnBPA expression on the outside bacterial membrane can be related to an improved internalization ability in eukaryote cells *in vitro*.

### *In vivo* invasiveness assay in the intestinal tract of mice

In an attempt to compare the DNA transfer properties *in vivo* of LL-FnBPA and LL-pNisFnBPA, the pValac plasmid containing the *gfp* gene [7] was used to transform these strains, thus obtaining strains denominated LL-FnBPA-GFP and LL-pNisFnBPA-GFP, respectively.

The subsequent oral administration of mice with LL-pOri23-GFP, LL-FnBPA-GFP or LL-pNisFnBPA-GFP allowed us to evaluate the effect of FnBPA expression on DNA delivery as observed by the presence of fluorescence (GFP expression by pValac:GFP) in the small and large intestine tissues, 24 hours after administration.

**Figure 3:**
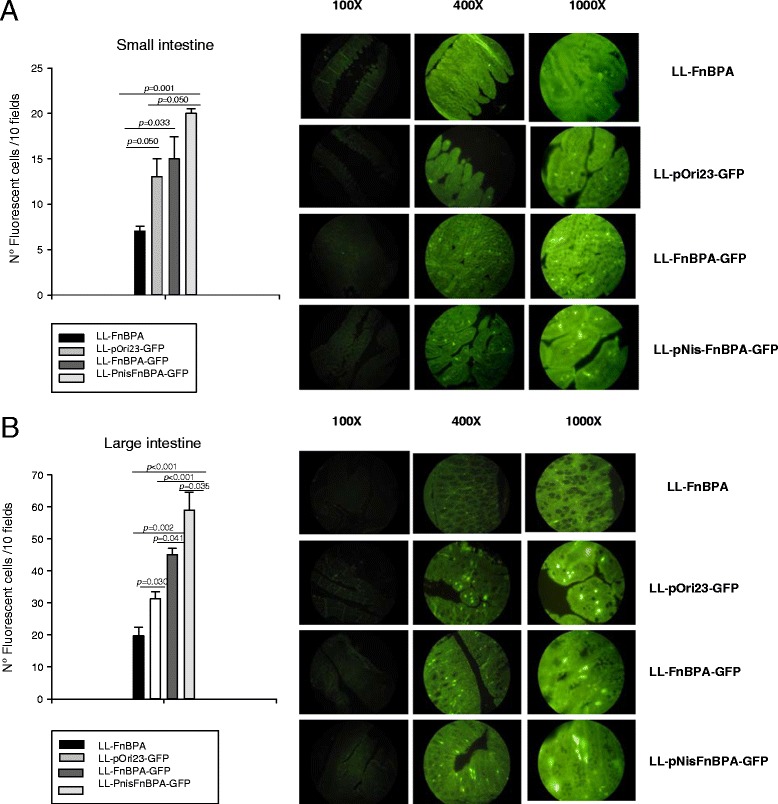
**Number of fluorescent cells in the A) small or B) large intestines of mice orally administered with LL-pNisFnBPA-GFP, LL-FnBPA, LL-pORI-GFP, LL-FnBPA-GFP.** On the left is represented the number of fluorescent cells determined in 10 fields of vision and when a significant difference was observed, the p values were places. On the right are representative histological slides observed at different magnifications using a fluorescent microscope.

The importance of FnBPA in the DNA delivery was observed mainly in large intestine. Mice that received LL-FnBPA-GFP showed significant increases (P < 0.05) in the number of fluorescent cells compared to the mice that received LL-pOri23-GFP (Figure 3). The improvement of the delivery system with the addition of the promoter pNisA to increase the expression of FnBPA was also demonstrated. Mice that received LL-pNisFnBPA-GFP strain showed the highest number of GFP positive cells in both small and large intestines (Figure 3); however, when comparing this strain with LL-FnBPA-GFP, a significant higher number of fluorescent cells was (P < 0.05) only observed in the large intestine (Figure 3).

Fluorescent cell counts are in favor of a more efficient DNA transfer associated with the LL-pNisFnBPA-GFP strain, where the FnBPA gene is under the control of the *nisA* promoter, compared with the native promoter LL-FnBPA-GFP strain, and the control LL-pOri23-GFP strain without FnBPA.

## Discussion

In this study, the design of a particular *Lactococcus lactis* strain expressing FnBPA on the external membrane under the control of the strong inducible *nisA* promoter was described. This so-called LL-pNisFnBPA strain was studied for its properties to improve DNA delivery *in vitro* on eukaryote cell models, as well as *in vivo* in mice intestinal tract. This recombinant strain was obtained from the transformation of the wild type *L. lactis* NZ9000 strain, since it contains the *nisR* and *nisK* genes which are needed for the regulation of the expression system based on the *nisA* promoter [18,19].

A sub-inhibitory nisin concentration (10 ng/ml) was used for the overnight induction of the LL-pNisFnBPA-GFP strain allowing then a 55% gain regarding FnBPA expression compared with LL-FnBPA where FnBPA production is under the control of its constitutive promoter. This shift is in agreement with the *nisA* promoter strength already described where the efficiency of the *nisA* promoter is compared to other promoter in Gram-positive bacteria [17].

It is now well established that LL-FnBPA recombinant strains exhibit good properties of internalization in Caco-2 cells as can be observed by GFP expression plasmid delivery [20] or through the expression of BLG *in vivo* [21].

Here, we confirmed this capacity *in vitro* on Caco-2 cells and showed in addition that an enhancement of FnBPA expression, by the use of LL-pNisFnBPA strain, resulted in an increase of invasiveness level of 4 to 5 times compared to the LL-FnBPA strain.

Moreover, we showed for the first time that FnBPA production at the surface of LAB increase invasiveness in BMDCS. Both strains LL-FnBPA and LL-pNisFnBPA showed higher invasiveness in BMDCs than in Caco-2 cells compared to non-invasive LL strain. This observation can come close to the fact that pathogenic bacteria uptake by dendritic cells also involves the DC maturation stage independently of the invasin [22]. The increase of FnBPA expression is directly related to the higher invasiveness level associated to the LL-pNisFnBPA strain when co-incubated with Caco2 cells or BMDCs.

An *in vivo* study was also performed (Figure 3) using the pValac delivery system with GFP being used as the indicator of DNA delivery and expression. The effectiveness of this DNA delivery system was demonstrated previously in healthy mice that received LL-FnBPA-GFP [21] and also in a TNBS-induced colitis mouse model, where the delivery of IL-10 DNA was evaluated using LL-FnBPA-IL10 [23]. In both animal models, the recombinant *L. lactis* strain expressed FnBPA under its own constitutive promoter. Here, it was observed that the expression of FnBPA under *Nis*A promoter increased the DNA delivery in the intestinal tract of mice that received LL-pNisFnBPA-GFP, compared to the mice that received LL-FnBPA-GFP.

The transit of *L. lactis* in the digestive tract has been especially described [24]. It turns out that *L. lactis* does not colonize the gastrointestinal tract and exhibits a low survival in the gut. As a matter of fact, if *L. lactis* is resistant to gastric acidity it can be noted that less than one third of the orally administrated bacteria survive in the duodenum. So, the expression of FnBPA can help to the DNA delivery by this LAB. This was confirmed in the present work where we detected increased DNA transfer in the cells of small intestine, and even more in the large intestine of mice following the administration of recombinant *L. lactis* strains that expressed FnBPA (LL-pNisFnBPA-GFP and LL-FnBPA-GFP), compared to the animals that received *L. lactis* without the fnbpa gene (LL-pOri23-GFP). It is also important to note that DNA delivery in the intestine was increased by the use of pNisA promoter in the recombinant strain as a replacement to the constitutive promoter used in other studies.

The animal model used in the present work did not show any adverse secondary effect in the mice that received the recombinant strains; however, considering that this experiment consisted in only 24 h, new experiments to evaluate the safety of this new recombinant strain are currently under way using healthy animals and also animals with some specific intestinal pathologies such as induced Crohn’s disease and colon cancer models.

## Conclusion

One of the immunotherapy research fields is looking for adapted bacterial vectors as DNA vaccine carriers [25-27]. In this report, we describe the design of a *L. lactis* recombinant strain where the expression of FnBPA, the invasin from *S. aureus*, is driven by the *nisA* promoter. We showed that this *L. lactis* strain provides an improved tool for bacterial invasiveness and DNA transfer as can be observed by co-incubation with Caco2 cells or BMDCs, as well as by GFP expression in small and large intestine after orally administration of recombinant strains that expressed pValac:*gfp.*

Therefore, these results highly suggest that the enhancement of FnBPA expression on the bacterial membrane through the engineering of a promoter system is directly correlated to the increase of DNA delivery properties, which is a good illustration of the knowledge acquired in the field of DNA vaccine or the production of other beneficial proteins.

## Methods

### Cloning and plasmid construction

The pSEC-E7 plasmid containing the *nisA* gene promoter was used to provide the corresponding fragment by digestion with *BamHI* and *BglII* enzymes. In addition the *BamHI* FnBPA gene fragment, without its constitutive promoter, was recovered by a *BamHI* digestion of a pORI23 plasmid containing the FnBPA gene (Table 1). This FnBPA fragment was then inserted in the pORI253 plasmid linearized with *Ecl136II*. The resulting construction was subsequently linearized by *SalI* digestion for the insertion of the *nisA* promoter upstream FnBPA to produce the Pnis-FnBPA plasmid. The cloning region was sequenced after each stage of the construction for orientation control.

**Table 1:** **Strains and plasmids used in this work along with their denomination in this work, mediums used for their growth and references**

**Host strain**	**Denomination**	**Plasmids**	**Medium and antibiotics**	**References**
NZ 9000	LL-NZ9000	No plasmid	M17 + 0.5% Glucose	[19]
MG1363	LL-FnBPA	pOri23 FnBPA	M17 + 0.5% Glucose, Ery ^R^	[16]
NZ9000	LL-pSEC-E7	pSEC-E7	M17 + 0.5% Glucose, Cm ^R^	[28]
NZ9000	LL-pNisFnBPA	pNis A - pOri253 FnBPA	M17 + 0.5% Glucose, Ery ^R^	This work
NZ9000	LL-pNisFnBPA-GFP	pNis A - pOri253 FnBPA	M17 + 0.5% Glucose, Ery ^R^ and Cm ^R^	This work
		pValac:GFP		
MG1363	LL-FnBPA-GFP	pOri23 FnBPA	M17 + 0.5% Glucose, Ery ^R^ and Cm ^R^	[20]
		pValac-GFP		
MG1363	LL-pOri23-GFP	pOri23	M17 + 0.5% Glucose, Ery ^R^ and Cm ^R^	This work
		pValac-GFP		
MG1363	LL-MG1363	No plasmid	M17 + 0.5% Glucose	[29]

### Cells

Human colon carcinoma cell line (Caco-2, ATCC), were routinely grown in 25 cm^2^ plastic flask (Costar) in DMEM, 10% SBF, 2 mM L-glutamine, 0.1 mg/L streptomycin, 100.000 U/L penicillin and 1 mM sodium pyruvate. Cells were kept at 37°C in 5% CO_2_, 90-95% humidity, trypsinized weekly (0.25% trypsin, 5 mM EDTA in Ca-Mg-Free Hanks’ balanced salt) and seeded onto new flask (1/10, v/v). Murine bone marrow dendritic cells (BMDCs) were generated from bone marrow (BM) progenitor cells isolated from femurs and tibias of BALB/c mice as previously described [30], with minor modifications. Briefly, after red cell lysis (ammonium chloride 0.14 M, pH 7.2), BM cells were cultured in Petri dishes at 2 × 10^5^ cells/mL in complete Iscove’s Modified Dulbecco’s Medium (IMDM, Sigma, St. Louis, USA) supplemented with 10% (v/v) heat-inactivated fetal calf serum (FCS, Gibco-BRL, Paisley, Scotland), 50 μM 2-mercaptoethanol, 1 mM glutamine, 50 μg/mL gentamycin and 20 ng/mL of recombinant mouse GM-CSF. Freshly prepared medium was added every three days and BMDCs were used on day 11 of culture (maximum of CD11c expression as checked by FACS analysis).

### Bacterial strains

*L. lactis* strains were cultured in M17 medium containing 0.5% glucose (GM17) at 30°C without shaking. Plasmids containing pNisA-FnBPA genes were transferred to *L. lactis* NZ 9000 by electroporation as previously described [31,32]. After preliminary tests, pValac-GFP plasmids were also introduced in *L. lactis* containing pNisA-FnBPA by the same way. Antibiotics were added at the indicated concentrations as necessary (erythromycin 5 μg/mL, chloramphenicol 10 μg/mL). Strains and plasmids used in this work are listed in Table 1.

### FnBPA induction and protein measurement

All experiment with the strain containing the *fnbpA* gene under the control of pNisA was performed using nisin (10 ng/mL; Sigma) as previously described [33] with some modifications. Briefly, overnight cultures in GM17 medium were diluted 1:20 and bacteria were grown until they reached an OD_600_ = 0.5, then nisin was added and bacteria grew for another 16 hours. To compare FnBPA levels, each strain was washed and resuspended in PBS, 1 × 10^5^ CFU were incubated with 500 μL of primary antibody (1/1000 anti-FnBPA α produced in rabbit [20] for 1 hour at 4°C. Pellet was washed with phosphate buffered saline (PBS) solution 0.01 M, pH 7.4 and the secondary antibody was added (1/100 - FITC conjugated affinity pure Fab fragment goat anti-rabbit IgG (H + L)) followed by incubation at 4°C during 1 hour. Samples were washed again and analyzed by flow cytometry (BD Accuri™ Flow Cytometer). Data were analyzed using CFlow Plus software.

### *In vitro* invasiveness assays of FnBPA strains in caco-2 and BMDCs

*In vitro* invasiveness assays were performed as previously described [5,7,34] using the human colon carcinoma cell line (Caco-2) or murine bone marrow dendritic cells (BMDCs). Cells were cultured as described above, and distributed in P12 dishes (4 × 10^5^ cells per well), bacteria were added to mammalian cells with a multiplicity of infection (MOI) of 10^3^ bacteria per cell in medium without antibiotics. After two hours of co-incubation, cells were carefully washed three times with PBS and treated with gentamicin (150 μg/mL) for two hours in order to kill extracellular bacteria. Cells were washed again and disrupted (sterile PBS 1X + 0.2% triton, 4°C during 10 minutes), colony forming units (CFU) were counted after plating serial dilutions of these cells. All experiments were done in triplicate.

### GFP detection in cells from the small and large intestines of mice

Bacterial strains were grown and prepared as described above with 10 ng/ml of nisin for induction in all strains (which is a sub inhibitory concentration but induces expression in the pNisFnBPA containing strain). LL-FnBPA was used as control without GFP; LL-pOri23-GFP and LL-FnBPA-GFP were used to evaluate the possible benefit in DNA delivery related to FnBPA expression, and LL-pNisFnBPA-GFP was used to examine the effect in DNA delivery related to increased bacterial invasiveness. Six-week-old female BALB/c mice weighing 20–25 g were obtained from the inbred closed colony maintained at CERELA (Centro de Referencia para Lactobacilos, San Miguel de Tucumán, Argentina). Three mice per group were fed by gavage with each bacterial suspension (10^8^UFC/mouse) orally using a gavage syringe. After 24 h of administration, mice were sacrificed by cervical dislocation and small and large intestines were removed, washed with PBS and prepared for histological evaluation using standard techniques. Serial paraffin sections (4 μm) were made and after deparaffinization were mounted in glycerol/PBS (9/1) solution and observed using a fluorescence light microscope. The number of fluorescent cells was counted by two different researchers (two individual blind counts per sample) and the results were expressed as the mean number of positive cells counted in ten fields of vision as seen at 1000 × magnification.

### Statistical analysis

Fluorescent intensity was analyzed using data from Accuri – CFlow Plus and GraphPad Prism 5.00 (Unpaired *t* test).

Invasiveness assays were also analyzed by means of and GraphPad Prism 5.00 using Bonferroni’s Multiple Comparison Test.

For animal trials, the test and control groups contained 3 animals and the experimental protocol was performed two times (no interactions between these two trials were observed) and all the results (from the two trials) were analyzed together (N = 6).

Statistical analyses were performed using MINITAB 15 software. Comparisons were accomplished by an ANOVA general linear model followed by a Tukey’s post hoc test.

### Ethics statement

The Animal Protection Committee of CERELA preapproved all animal protocols and all experiments complied with the current laws of Argentina.

## Abbreviations

FnBPA: Fibronectin Binding Protein A
LL: Lactococcus lactis
BMDC: Bone marrow dendritic cell
MOI: Multiplicity of infection

## References

[1] Bermudez-Humaran LG, Kharrat P, Chatel JM, Langella P (2011). Lactococci and lactobacilli as mucosal delivery vectors for therapeutic proteins and DNA vaccines. Microbiol Cell Factories.

[2] Wells JM, Mercenier A: **Mucosal delivery of therapeutic and prophylactic molecules using lactic acid bacteria.** *Nature Rev Microbiol* 2008, doi:10.1038/nrmicro1840. 10.1038/nrmicro1840 PMC7096801 18345021

[3] Chatel JM, Pothelune L, Ah-Leung S, Corthier G, Wal JM, Langella P (2008). *In vivo* transfer of plasmid from food-grade transiting lactococci to murine epithelial cells. Gene Ther.

[4] Dellaretti Guimaraes V, Innocentin S, Lefèvre F, Azevedo V, Wal JM, Langella P, Chatel JM (2006). Use of native lactococci as vehicles for delivery of DNA into mammalian epithelial cells. Appl Environ Microbiol.

[5] Dellaretti Guimaraes V, Gabriel JE, Lefèvre F, Cabanes D, Gruss A, Cossart P, Azevedo V, Langella P (2005). Internalin-expressing Lactococcus lactis is able to invade small intestine of guinea pigs and deliver DNA into mammalian epithelial cells. Microb Infect.

[6] Pontes DS, De Azevedo MS, Chatel JM, Langella P, Azevedo V, Miyoshi A (2011). *Lactococcus lactis* as a live vector : heterologous protein production and DNA delivery systems. Protein Expr Purif.

[7] Guimaraes V, Innocentin S, Chatel JM, Lefevre F, Langella P, Azevedo V, Miyoshi A (2009). A new plasmid vector for DNA delivery using lactococci. Genet Vaccines Ther.

[8] Cossart P, Sansonetti PJ (2004). Bacterial invasion: the paradigms of enteroinvasive pathogens. Science.

[9] Sinha B, François P, Que YA, Hussain M, Heilmann C, Moreillon P, Lew D, Krause KH, Peters G, Herrmann M (2000). Heterologously expressed *Staphylococcus aureus* fibronectin-binding proteins are sufficient for invasion of host cells. Infect Immun.

[10] Gaillard JL, Berche P, Frehel C, Gouin E, Cossart P (1991). Entry of *L. monocytogenes* into cells is mediated by internalin, a repeat protein reminiscent of surface antigens from gram-positive cocci. Cell.

[11] Lecuit M, Ohayon H, Braun L, Mengaud J, Cossart P (1997). Internalin of *listeria monocytogenes* with an intact leucine-rich repeat region is sufficient to promote internalization. Infect Immun.

[12] Dersch P, Isberg RR (1999). A region of the *yersinia pseudotuberculosis* invasin protein enhances integrin-mediated uptake into mammalian cells and promotes self-association. EMBO J.

[13] Hamburger ZA, Brown MS, Isberg RR, Bjorkman PJ (1999). Crystal structure of invasion: a bacterial integrin-binding protein. Science.

[14] Schwarz-Linek U, Höök M, Potts JR (2004). The molecular basis of fibronectin-mediated bacterial adherence to host cells. Mol Microbiol.

[15] Que YA, François P, Haefliger JA, Entenza JM, Vaudaux P, Moreillon P (2001). Reassessing the role of *staphylococcus aureus* clumpin factor and fibronectin-binding protein by expression in *lactococcus lactis*. Infect Immun.

[16] Que YA, Haefliger JA, Piroth L, François P, Widmer E, Entenza JM, Sinha B, Herrmann M, Francioli P, Vaudaux P, Moreillon P (2005). Fibrinogen and fibronectin binding cooperate for valve infection and invasion in *staphylococcus aureus* experimental endocarditis. J Exp Med.

[17] Eichenbaum Z, Federle MJ, Marra D, De Vos WM, Kuipers OP, Kleerebezem M, Scott JR (1998). Use of the lactococcal *nisA* promoter to regulate gene expression in gram-positive bacteria: comparison of induction level and promoter strength. Appl Environ Microbiol.

[18] Mierau I, Kleerebezem M (2005). 10 years of the nisin-controlled gene expression system (NICE) in *lactococcus lactis*. Appl Microbiol Biotechnol.

[19] Kuipers CP, Beerthuyzen MM, De Ruyter PG, Luesink EJ, De Vos WM (1995). Autoregulation of nisin biosynthesis in *lactococcus lactis* by signal tranduction. J Biol Chem.

[20] Innocentin S, Guimaraes V, Miyoshi A, Azevedo V, Langella P, Chatel JM, Lefèvre F (2009). *Lactococcus lactis* expressing either *staphylococcus aureus* fibronectin-binding protein A or *listeria monocytogenes* internalin a can efficiently internalyze and deliver DNA in human epithelial cells. Appl Environ Microbiol.

[21] Pontes D, Innocentin S, Del Carmen S, Almeida JF, LeBlanc JG, De Moreno de LeBlanc A, Blugeon S, Cherbuy C, Lefèvre F, Azevedo V, Miyoshi A, Langella P, Chatel JM (2012). Production of fibronectin binding protein A at the surface of *lactococcus lactis* increases plasmid transfer *in vitro* and *in vivo*. PlosOne.

[22] Kolb-Maurer A, Gentschev I, Fries HW, Fiedler F, Bröcker EB, Kämpgen E, Goebel W (2000). *Listeria monocytogenes*-infected human dendritic cells: uptake and host cell response. Infect Immun.

[23] Del Carmen S, Zurita-Turk M, Alvarenga Lima FA, Coelho Dos Santos JS, Leclercq SY, Chatel JM, Azevedo V, De Moreno De LeBlanc A, Miyoshi A, LeBlanc JG (2013). A novel interleukin-10 DNA mucosal delivery system attenuates intestinal inflammation in a mouse model. Eur J Inflam.

[24] Drouault S, Corthier G, Ehrlich D, Renault P (1999). Survival, physiology and lysis of *lactococcus lactis* in the digestive tract. Appl Environ Microbiol.

[25] Schoen C, Stritzker J, Goebel W, Pilgrim S (2004). Bacteria as DNA vaccine carriers for genetic immunization. Int J Med Microbiol.

[26] Becker PD, Noerder M, Guzman CA (2008). Genetic immunization. Human Vaccines.

[27] De Azevedo MSP, Innocentin S, Dorella FA, Rocha CS, Mariat D, Pontes DS, Miyoshi A, Azevedo V, Langella P, Chatel JM: **Immunotherapy of allergic diseases using probiotics or recombinant probiotics.** *J Appl Microbiol* 2013, doi:10.1111/jam.12174. 10.1111/jam.12174 23437848

[28] Bermudez-Humaran LG, Cortes-Perez NG, Le Loir Y, Gruss A, Rodriguez-Padilla C, Saucedo-Cardenas O, Langella P, Montes de Oca-Luna R (2003). Fusion to a carrier protein and a synthetic propeptide enhances E7 HPV-16 production and secretion in *lactococcus lactis*. Biotechnol Prog.

[29] Gasson MJ (1983). Plasmid complements of Streptococcus lactis NCDO 712 and other lactis streptococci after protopalst-induced curing. J Bacteriol.

[30] Lutz MB, Kukutsch N, Ogilvie AL, Rössner S, Koch F, Romani N, Schuler G (1999). An advance culture method for generating large quantities of highly pure dendritic cells from mouse bone marrow. J Immunol Methods.

[31] Bermudez-Humaran LG, Langella P, Cortes-Perez NG, Gruss A, Tamez-Guerra RS, Oliveira SC, Saucedo-Cardenas O, Montes de Oca-Luna R, Le Loir Y (2003). Intranasal immunization with recombinant *lactococcus lactis* secreting murine interleukin-12 enhances antigen-specific Th1 cytokine production. Infect Immun.

[32] Cortes-Perez NG, Bermudez-Humaran LG, Le Loir Y, Rodriguez-Padilla C, Gruss A, Saucedo-Cardenas O, Langella P, Montes-de-Oca-Luna R (2003). Mice immunization with live lactococci displaying a surface anchored HPV-16 E7 oncoprotein. FEMS Microbiol Lett.

[33] Enouf V, Langella P, Commissaire J, Cohen J, Corthier G (2001). Bovine rotavirus nonstructural protein 4 produced by *lactococcus lactis* is antigenic and immunogenic. Appl Environ Microbiol.

[34] Dramsi S, Biswas I, Maguin E, Braun L, Mastroeni P, Cossart P (1995). Entry of listeria monocytogenes into hepathocytes requires expression of inlB, a surface protein of the internalin multigene family. Mol Microbiol.

